# Herbivory in a changing climate—Effects of plant genotype and experimentally induced variation in plant phenology on two summer‐active lepidopteran herbivores and one fungal pathogen

**DOI:** 10.1002/ece3.8495

**Published:** 2022-01-25

**Authors:** Adam Ekholm, Maria Faticov, Ayco J. M. Tack, Tomas Roslin

**Affiliations:** ^1^ Department of Ecology Swedish University of Agricultural Sciences Uppsala Sweden; ^2^ Department of Ecology Environment and Plant Sciences, Stockholm University Stockholm Sweden

**Keywords:** Climate change, Community ecology, Mismatch, Phenology, Trophic interactions

## Abstract

With climate change, spring warming tends to advance plant leaf‐out. While the timing of leaf‐out has been shown to affect the quality of leaves for herbivores in spring, it is unclear whether such effects extend to herbivores active in summer. In this study, we first examined how spring and autumn phenology of seven *Quercus robur* genotypes responded to elevated temperatures in spring. We then tested whether the performance of two summer‐active insect herbivores (*Orthosia gothica* and *Polia nebulosa*) and infection by a pathogen (*Erysiphe alphitoides*) were influenced by plant phenology, traits associated with genotype or the interaction between these two. Warm spring temperatures advanced both bud development and leaf senescence in *Q*. *robur*. Plants of different genotype differed in terms of both spring and autumn phenology. Plant phenology did not influence the performance of two insect herbivores and a pathogen, while traits associated with oak genotype had an effect on herbivore performance. Weight gain for *O*. *gothica* and ingestion for *P*. *nebulosa* differed by a factor of 4.38 and 2.23 among genotypes, respectively. Herbivore species active in summer were influenced by traits associated with plant genotype but not by phenology. This suggest that plant attackers active in summer may prove tolerant to shifts in host plant phenology—a pattern contrasting with previously documented effects on plant attackers active in spring and autumn.

## Graphical abstract

We found that spring and autumn phenology differed among plant genotypes, but that neither the performance of the summer active herbivores nor the fungal pathogen were influenced by host plant phenology. This implies that herbivores active in summer can – at least to a certain extent – tolerate climate‐induced shifts in host plant phenology.

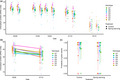

## 1 INTRODUCTION

In seasonal environments, organisms rely on cues to infer when conditions are favorable enough to become active, and on cues to decide when conditions are less favorable and activity should be reduced. One such cue is temperature, which plays a key role in the timing of several phenological events such as the bud burst of trees, the flowering of plants, the release of fungal spores, the emergence of insects, and the senescence of leaves (Fu et al., 2018; Marcais et al., 2009; Menzel et al., 2006; Roy & Sparks, 2000; Zohner & Renner, 2019). At present, temperatures are increasing, and will become even higher in the future (IPCC, 2014). In response to such warmer temperatures, many organisms are expected to advance their spring phenology, and the rate of advance can differ among species (Kharouba et al., 2018; Thackeray et al., 2016). Such changes in phenology can influence the interaction strengths among species (Kharouba et al., 2018; Liu et al., 2011; Meineke et al., 2021). In producer‐consumer interactions, the quality of a living resource may change with its phenological stage (Barton et al., 2019), which could result in changes in resource quality in producer‐consumer relationships.

In spring, warm temperatures usually advance phenology in plants (Fu et al., 2015; Piao et al., 2015; Zohner & Renner, 2019). In autumn, high spring temperatures can advance leaf senescence, whereas high summer and autumn temperatures can delay leaf senescence (Fu et al., 2014, 2018; Gill et al., 2015; Zohner & Renner, 2019). Many trees show genetically determined variation in phenology (Vitasse et al., 2013). Yet, plant genotypes can differ in a wealth of other traits, including leaf nitrogen levels, defensive compounds, and other traits (Barbour et al., 2015; Falk et al., 2018; Vitasse et al., 2013). Such differences may translate into shifting performances of insect herbivores and pathogens attacking different genotypes (Barker et al., 2019; Ekholm et al., 2017; Falk et al., 2018). However, with elevated temperature and changing phenologies, plant attackers that currently overlap in time with leaf development of certain plant genotypes might slide out of synchrony and become better synchronized with other genotypes (Genotype by Environment interaction). Changes in the relative phenology of genotypes within populations could shift the strength and distribution of pairwise interactions between plant attackers and specific host plant genotypes.

Shifts in synchrony between interacting species may be particularly important for deciduous trees and their consumers. When new leaves are produced in spring, the physical and chemical properties of a leaf change rapidly after bud burst. Fresh leaves are usually dominated by high levels of nitrogen and defensive compounds, while old leaves have a tougher surface than fresh leaves (Barton et al., 2019; Falk et al., 2018; Feeny, 1970; Salminen et al., 2004). Studies have demonstrated that leaf age can have an effect on both survival and growth rate of insect herbivores and infection by pathogens, where young leaves are usually favored (Barbehenn et al., 2017; Dantec et al., 2015; Dodd et al., 2008; Edwards & Ayres, 1982; Falk et al., 2018; Tikkanen & Julkunen‐Tiitto, 2003). From the perspective of a plant attacker, rapid changes in leaf properties can define a “window of opportunity” in spring, where leaves of a certain age are of particularly high quality to the attacker. The timing of leaf senescence can also be of great importance for plant attackers active late in the season. For late‐season plant attackers, a tree that withdraws nutrients from its leaves (Maillard et al., 2015) early in autumn will probably provide a lower‐quality resource in comparison to a tree that maintains nutrients in the leaf for a longer time. Such variation in autumn phenology can have an influence on species abundances (Ekholm et al., 2019).

**FIGURE 1:**
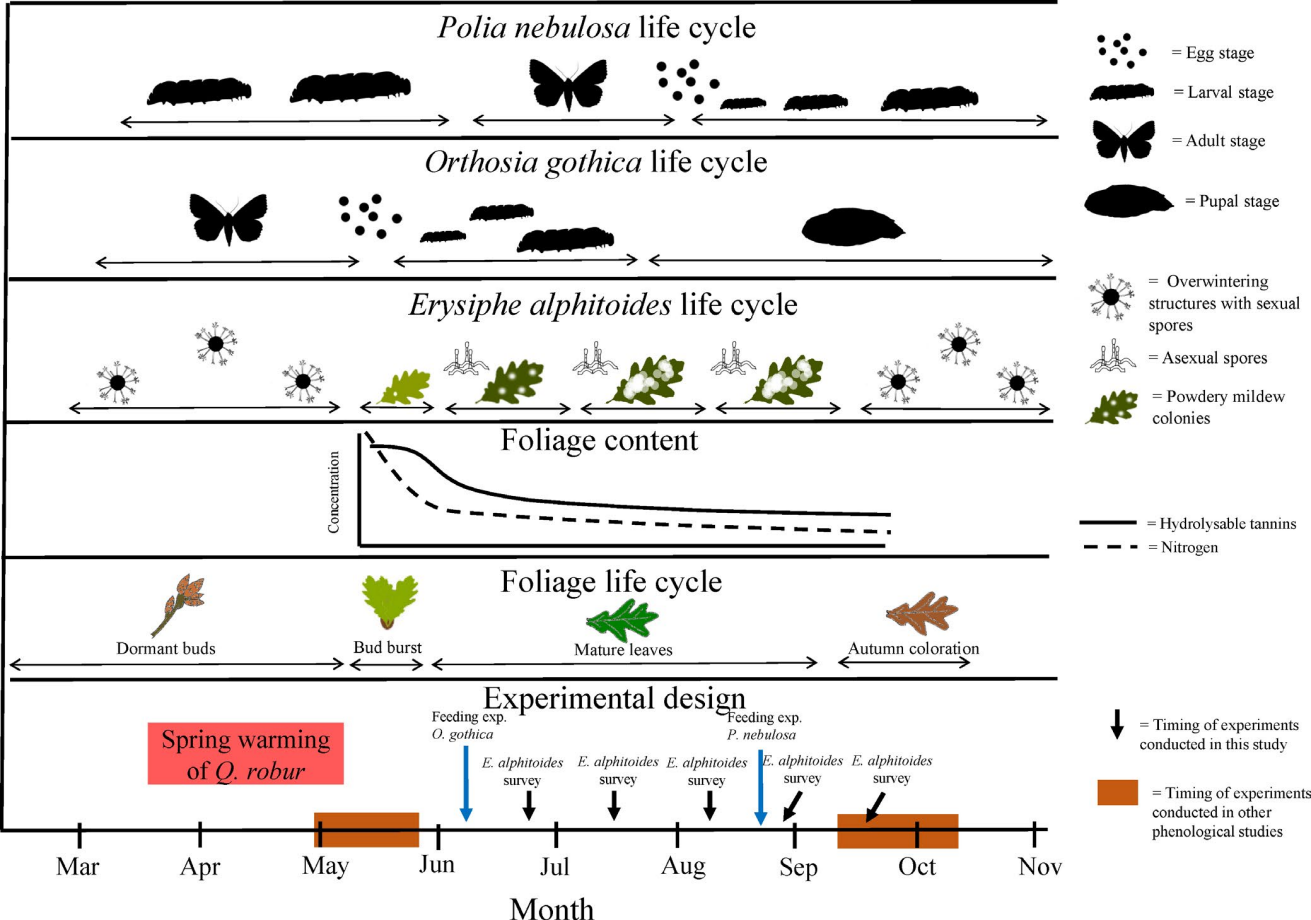
A schematic illustration of the temporal context of the study. At the bottom of the figure is the timing of previous phenological studies shown with brown boxes, while the timing of this study is shown with blue arrows, as compared to known patterns in the chemical contents of oak leaves (here represented by nitrogen and hydrolyzable tannins at the fourth row, from Salminen et al. (2004)). As can be seen, previous studies have focused on herbivore performance during short time windows in spring (Falk et al., 2018; Tikkanen & Julkunen‐Tiitto, 2003) and autumn (Connor et al., 1994; Ekholm et al., 2019; Mopper & Simberloff, 1995), whereas few if any studies have focused on the effect of plant phenology on herbivores active in the summer – despite hypothesized effects of leaf age. In this study, we focus on how the performance of two herbivores active during different parts of the summer are influenced by plant phenology. To establish phenological variation within a set of oak trees, we advance the phenology in half of our trees by exposing them to elevated temperatures in early spring (red box). In addition, we follow the development of a plant pathogen on plants of different phenology throughout the season. Consequences of leaf age were assessed both by snap‐shot bioassays conducted on June 6–8 (for *O*. *gothica*) and August 21–23 (for *P*. *nebulosa*) and by repeated surveys of *E*. *alphitoides* incidence on five specific dates (June 30, July 16, August 3, August 27, September 16). To illustrate the timing of experimental treatments and assays versus the life cycle of consumer species, we indicate the timing of different life stages by plants and plant attackers in the figure

While plant phenology in both spring and autumn can impact plant attackers, less is known whether variation in leaf age during summer also affect the performance of plant consumers. Changes in leaf chemistry is rapid in spring, with a reduction in both nitrogen and defensive compounds (Barton et al., 2019; Falk et al., 2018; Salminen et al., 2004; Zidorn, 2018). Such changes continue in early summer, but at a lower rate than in the spring (Figure 1; Riipi et al., 2002; Salminen et al., 2004; Zidorn, 2018). In the case of *Quercus robur*, hydrolyzable tannins are an important group of defensive compounds that decrease at a rapid rate in the early season and then levels off toward the end of the season. However, individual compounds vary in their seasonal patterns (Salminen et al., 2004). Similarly, nitrogen content continues to drop over the growing season. Thus, phenology‐mediated differences in nitrogen and defensive compounds among trees are likely to persist into the summer—but being less pronounced than in spring. It is thus important to understand whether herbivores active in summer are affected by changes in the leaf‐out date in the spring. While variation in bud break might affect the performance of plant consumers during summer, and affect resource quality from the insect's perspective, the extent of such effects remain to be established.

To assess how plant phenology responds to a warmer climate, and whether such changes influence plant attackers active in the summer, we focused on the oak *Quercus robur* and its associated taxa. In spring, we experimentally manipulated temperature and scored the phenology of seven *Q*. *robur* genotypes. We then investigated how this temperature‐induced variation in host plant phenology interacted with host plant genotype in determining leaf quality from the insect's perspective (assessed through bioassays of herbivore performance). Given the many types of changes that may occur in a growing leaf (Salminen et al., 2004) and their differential effects on different organisms (Roslin & Salminen, 2008; Schoonhoven et al., 2005), we explicitly focused on concrete bioassays of performance rather than chemical characterization. Targeting the performance of two insect herbivores and a specialist fungal pathogen feeding on oak leaves, we specifically asked:

- From the perspective of the tree (*Q*. *robur*):
  - Does the effect of spring‐time temperatures on spring and autumn phenology differ among *Q*. *robur* genotypes?
- From the perspective of plant attackers
  - Does host plant phenology and other traits associated with host plant genotype influence the performance of free‐feeding lepidopteran larvae active in summer?
  - Does host plant phenology and other traits associated with host plant genotype influence the performance of a fungal pathogen?

## 2 MATERIAL AND METHODS

### 2.1 Study system

Oaks (genus *Quercus*) are widely distributed across several continents (Denk et al., 2017). Within Europe, *Q*. *robur* is a widespread and locally common species (Eaton et al., 2016), which—compared to other trees—hosts a particularly rich community of insects (Southwood, 1961). Phenology in *Q*. *robur* varies over a latitudinal gradient (Ekholm et al., 2019) and can differ by *c*. 20 days within populations (Delpierre et al., 2017).

The insect community associated with *Q*. *robur* has been particularly well studied (Ekholm et al., 2019; Kaartinen et al., 2010; Stone et al., 2002). Of the herbivore species found on *Q*. *robur*, the free feeding larvae of *Orthosia gothica* (Lepidoptera: Noctuidae) and *Polia nebulosa* (Lepidoptera: Noctuidae) are two generalist herbivores that occur over a large part of Europe (Elmquist et al., 2011; Karsholt & Razowski, 1996). These species have different life cycles. *O*. *gothica* overwinters as pupae, adults emerge and oviposit in spring, and larvae feed on foliage at the start of the growing season (Figure 1; Elmquist et al., 2011; Pöyry et al., 2018). In contrast, *P*. *nebulosa* overwinters as larvae and adults are active in summer (Elmquist et al., 2011), with larvae feeding on leaves from August and again in spring next year (Figure 1; Svensson, 1993). Another locally common herbivore is the leaf miner *Acrocercops brongniardellus* (Lepidoptera: Gracillariidae). It is a spring‐active oak specialist that is distributed over a large part of Europe (Karsholt & Razowski, 1996). It attacks newly flushed leaves and can sometimes reach outbreak densities. At outbreak densities, up to 90% of the leaves on a single oak can be attacked and a large part of the leaf is usually consumed (Bengtsson & Johansson, 2011; Ekholm et al., 2020).

Apart from insect herbivores, several fungal pathogens are also known to attack *Q*. *robur*. One of the fungal pathogens specializing on oaks is *Erysiphe alphitoides* (Erysiphales: Erysiphaceae). *E*. *alphitoides* grows on the leaf surface and the white appearance of its mycelium and conidial spores makes this pathogen easy to identify in the field (Woodward et al., 1929). The main mode of leaf infection in spring is thought to be by the release of sexual spores (ascospores) from overwintering structures called chasmothecia, which are produced on senescing leaves during the previous autumn (Figure 1; Marcais et al., 2009). The pathogen produces multiple generations of conidial (asexual) spores throughout the growing season (Figure 1).

### 2.2 Experimental design

To generate a set of replicate trees of known genotype, we established a set of grafted oak trees by propagating twigs from seven mother trees growing in a 5 km^2^ island in south‐western Finland. Grafting took place during 2011–2013. This was done by grafting each scion of given mother tree onto a separate randomly selected root stock of Finnish origin. The grafts first grown in a common garden and later (after one year) transferred to a 50 L pot. In this study, we utilized 160 of these grafted oaks (height of *c*. 1.5 m) from seven clones (henceforth referred to as genotypes). For a summary of the study design, see Figure 1.

#### 2.2.1 Effect of spring warming on spring phenology

In the spring of 2018, we manipulated the phenological variation among all grafted oaks by placing 77 oaks in a greenhouse with slightly elevated temperature in spring (average of 5°C warmer), while the remaining 83 oaks were subjected to ambient temperature in the field. Individuals of all genotypes were represented in both temperature treatments. As there was a slight difference in height among oaks, we used constrained randomization to allow for a similar average height in both treatments. Since the local temperature can vary within a greenhouse, we randomized each oak to a new position every week. The oaks subjected to ambient temperature were placed in an open field to mimic light conditions in the greenhouse. We also made sure that the soil humidity were kept at similar levels between treatments, by watering the oaks when necessary. Oaks from both treatments were moved to the field site in Länna (Sweden; 59.87° N, 17.96° E) between May 2 and 4, when oak trees in the greenhouse treatment initiated spring development (i.e., when buds were elongating and the tip of first leaves were visible). At the field site, we placed the 160 oak trees within five blocks, with 28–35 oaks per block. Within blocks, we randomized the position of each oak. Oaks were watered on a regular basis at the field site.

Initially, all seven genotypes subjected to both treatments were represented in each block. However, seven oaks died or did not develop well during the experiment, which reduced the number of oaks on which autumn phenology was scored (Table S1).

To study whether bud development in *Q*. *robur* was influenced by spring temperature and oak genotype, we scored bud development at the shoot level (according to Hinks et al., 2015; Table S2). We recorded phenology on 1–5 shoots per tree by scoring the median development stage of each shoot. Spring phenology was scored every 3rd to 4th day from April 26 until May 23, with a final survey at May 30.

#### 2.2.2 Effect of spring warming on autumn phenology

To assess whether autumn phenology was influenced by oak genotype and spring phenology, we surveyed the autumn phenology of two randomly selected leaves per shoot (five shoots per oak) by measuring chlorophyll content and estimating autumn leaf coloration. To obtain a rough estimate of chlorophyll content, we used a SPAD‐502 chlorophyll content meter (Spectrum Technologies Inc., Plainfield, IL, USA). To reduce measurement noise, we took five measurements per leaf and used the averaged value for the analyses. Leaf autumn color was scored on a binary scale: 0 = ≥90% of the leaf is green; 1 = <90% of the leaf is green. The latter cut‐off level was chosen to reflect a threshold that was easy to score and signaled the initiation of autumn leaf coloration. A threshold of 90% resulted in a larger variation in the binary response variable in comparison to using other thresholds. Autumn phenology was scored on August 30, September 12, September 19, October 1, and October 15.

#### 2.2.3 Herbivore performance

For metrics of leaf quality, we explicitly took an organism‐centered approach. Rather than measuring a wealth of individual chemical parameters, we explicitly focused on bioassays of herbivore performance. To investigate how traits associated with oak genotype and spring warming influenced such performance, we conducted two separate feeding experiments under laboratory conditions (Figure 1). In the first experiment (June 6–8), we used larvae of *Orthosia gothica* (*n* = 133) and in the second experiment (August 21–23), we used larvae of *Polia nebulosa* (*n* = 55). Prior to the experiments, we fed all larvae with a mixture of leaves from *Salix caprea* and *Quercus robur*. All larvae had been reared from eggs of known mothers (larvae from the same mother are defined as a brood), caught by hand netting or traps, and stored in plastic vials until oviposition took place. Thus, in each experiment, we measured the amount of ingested dry leaf mass and weight gain during 48 h—a time period sufficiently short to avoid larval abstinence from feeding just before and after moulting into a new instar.

Before we initiated each feeding experiment, we first chose a set of larvae of similar weight across larval families (*O*. *gothica*: range = 0.0141–0.0553 g, mean = 0.0303 g, SD = 0.0086 g; *P*. *nebulosa*: range = 0.0071–0.0361 g, mean = 0.0228 g, SD = 0.0068 g). We then randomly assigned each larva to a leaf collected from one of the grafted oak trees. Larvae and leaves were individually weighed prior to the experiment, then placed in a petri dish sealed by parafilm, and kept moist by a humid filter paper.

We placed each petri dish in a climate chamber with a temperature of 20°C, and a light cycle of 18 L: 6 D. To avoid any positional effects, we randomly changed the position of the petri dishes after c. 24 h. After c. 48 h, the petri dish was opened and the larvae and the leaf were weighed again. This allowed us to calculate larval weight gain and the fresh weight of ingested food. All larvae showing signs of moulting were removed from the experiment, since (as also stated above) they stop feeding at this stage.

To achieve a precise measure of the dry weight of ingested leaves, we separately estimated weight loss due to evaporation alone, using control leaves in individual petri dishes (Exp. 1, *n* = 9; Exp. 2., *n* = 4) with larvae excluded. In these leaves, we created a 1 cm cut in each leaf to mimic damage caused by a larva. These leaves were then dried in 50°C for at least 24 h. The weight loss to larval ingestion was then calculated using the following equation:

$$\text{Ingestion}=\frac{\text{Dry}\;\text{weight}}{\text{Fresh}\;\text{weight}}\times \text{Consumed}\;\text{fresh}\;\text{leaf}\;\text{mass}$$

where the Dry/Fresh weight ratio is calculated from the control leaves.

#### 2.2.4 Pathogen infection

To examine if traits associated with plant genotype and spring temperature treatment influenced *Erysiphe alphitoides*, we recorded incidence of *E*. *alphitoides* on five specific dates (June 30, July 16, August 3, August 27, September 16). On each tree, we visually scored the incidence of powdery mildew infection (i.e., presence of mycelium and spores on the upper leaf surface). *E*. *alphitoides* infection at the tree level was scored on a binary scale, where 0 = no signs of *E*. *alphitoides* infection, 1 = *E*. *alphitoides* present at least in small amounts.

### 2.3 Statistical analyses

All analyses below were performed in R (R Core Team, 2019). For ordinal responses, we used a cumulative link mixed model (CLMM) with a logit link from package *ordinal* (Christensen, 2018) and assessed significance with a likelihood ratio test using package *RVAideMemoire* (Maxime, 2019). For continuous or binomial responses, we used a linear mixed model or a generalized liner mixed model with a logit link from package *lme4* (Bates et al., 2015) or *nlme* (Pinheiro et al., 2019) and assessed significance with a type III ANOVA from the *car* package (Fox & Weisberg, 2019), or a marginal ANOVA from the *nlme* package (Pinheiro et al., 2019). If we detected pronounced heteroscedasticity when plotting the residuals against the predicted values, we estimated the variance separately by applying the function *varIdent* (Pinheiro et al., 2019) to one or several of the explanatory variables in the model. Model simplification was applied to all models below, where interactions for which *p* > .1 were removed from the final model. All models are summarized in Table S3.

During the experiment, there was an outbreak of the leaf‐mining species *A*. *brongniardellus* in the area, with the percentage of infested leaves varying from 0 to 93% per oak. Oak phenology and *A*. *brongniardellus* turned out to be highly correlated, which is shown in a different study (Ekholm et al., 2020). Here, we specifically adjust for potential effects of *A*. *brongniardellus* by including the fraction of leaves infested by this species (at the tree level) as a covariate in analyses of autumn phenology and fungal infection.

As two “nuisance” variables, we adjusted for effects of past tree damage and treatments received in prior experiments. With respect to the former, some (35 out 160) of the oak trees in this study appeared damaged in the tree nursery, as evidenced by dead branches or few opened buds. Tree damage status (no damage vs. damaged) was therefore included as a categorical fixed effect in all models below. With respect to treatments received in prior experiments, we note that a subset of the oaks had been part of a phenological experiment in the previous year (Faticov et al., 2020). We therefore tested for—but found no—spill‐over effects from temperature treatments in the preceding year on the spring phenology observed in the current experiment (Appendix B).

#### 2.3.1 Effect of spring warming on spring phenology

To assess whether oak spring phenology differed among oak genotypes and temperature treatments in 2018, we used recordings of spring phenology (ordinal metric, see above) on two dates: May 8 and 11. We then modeled shoot phenology at each date as a function of genotype, treatment in spring, and their interaction. To account for variation among blocks and oaks, we included block as a random effect and nested oak tree under block (model 1 in Table S3). Due to convergence issues, we removed the genotype with the fewest replicates (G5, *n* = 11 replicates unevenly distributed among blocks) from the analysis of responses scored on May 8.

#### 2.3.2 Effect of spring warming on autumn phenology

To detect whether oak autumn phenology was affected by oak genotype, date, and spring warming, we created several models. First, we examined if chlorophyll content differed among oak genotypes and treatments in spring by modeling chlorophyll content (a continuous metric) at each survey date (except October 15, as many leaves had dropped at this date) as a function of temperature treatment in spring and genotype (model 2 in Table S3). We estimated the variance separately for each treatment and genotype combination. Then, to assess if chlorophyll content differed between dates and if chlorophyll loss differed among genotypes, we modeled leaf chlorophyll content at two specific dates (September 19, October 1), as a function of oak genotype, date, and the interaction (model 4 in Table S3). The two dates represents the initiation of chlorophyll breakdown, as the latter date has lower chlorophyll content than all former survey dates (Figure 3). In this model, we estimated the variance separately for the different levels of genotype and tree quality. We averaged our leaf‐level observations of chlorophyll content across each oak × date combination in all models. In the last model, we modeled autumn leaf coloration at October 1 as our response. This date had the highest variation in autumn leaf coloration, where former and later dates were mostly dominated by green and brown leaves, respectively. We modeled autumn leaf coloration as a binary response, where we assigned oaks that had more than 50% autumn‐colored leaves (a leaf with <90% green color, see above) to one class and oaks with 50% or less autumn colors was assigned to the second class. We then modeled autumn leaf coloration as a function of temperature treatment in spring and genotype (model 4 in Table S3). In all models, we included block as a random effect to account for variation among blocks. When modeling chlorophyll content at two dates, we nested tree under block to account for measuring the same set of oak trees on two dates.

#### 2.3.3 Herbivore performance

To assess whether spring temperature treatment and traits associated with oak genotype affected the performance of free feeding lepidopteran larvae, we created two linear models for each specific experiment and modeled larval weight gain (Weight*_t_*_+48 h_–Weight*_t_*) and ingested leaf dry mass, respectively, as a function of temperature treatment in spring and oak genotype. As larvae differed in their initial weight and in what brood they came from, we included initial weight as a covariate and brood as a fixed effect. Here, the brood effect will capture multiple influences including genetic effects, but also maternal influences and impacts of the fact that siblings from the same broods were reared together, in one or several vials, until used in the experiment. When modeling ingestion in the first experiment (June 6–8), the two levels of tree damage status (no damage and damaged) differed in variance and we therefore fitted a separate variance to each level. Similarly, we fitted a separate variance to each oak genotype in the second experiment (August 21–23). See model 5–8 in Table S3.

#### 2.3.4 Pathogen infection

To determine how temperature treatment in spring, *A*. *brongniardellus* infestation and traits associated with oak genotype influenced infection by the pathogen *Erysiphe alphitoides*, we created date‐specific models and modeled incidence of *E*. *alphitoides* as a function of oak genotype, *A*. *brongniardellus* infestation, temperature treatment, and tree damage status. To account for variation among blocks, we included block as a random effect (model 9 in Table S3).

## 3 RESULTS

### 3.1 Effect of spring warming on spring phenology

**FIGURE 2:**
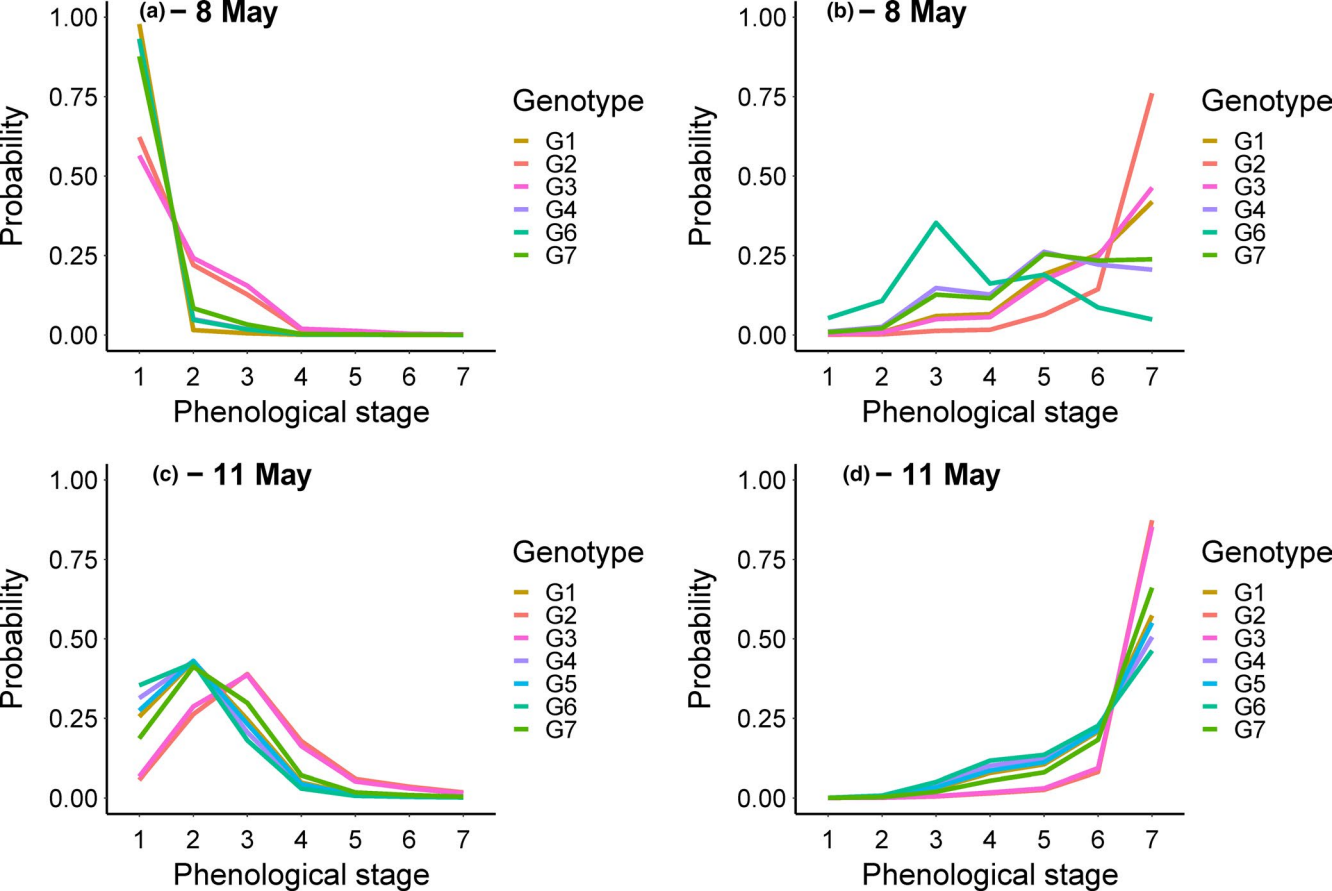
The probability of an oak tree to be in one out of seven different phenological stages in spring, where 1 represents a dormant bud and 7 a fully developed leaf. Shown are fitted values from different oak genotypes on two dates: May 8 (a, b) and May 11 (c, d), as split between oaks exposed to ambient (a, c) or elevated (b, d) temperatures in the spring

On both May 8 and 11, bud development was more advanced for oaks subjected to spring warming than for oaks maintained at ambient temperature, and also more advanced among trees without damage than damaged trees (Figure 2; Table 1). In addition, bud development differed among oak genotypes, although for the data of May 8, this effect varied among treatments (Figure 2; Table 1).

**TABLE 1:** The effect of spring warming (W), oak genotype (G), and tree damage status on spring and autumn phenology of *Q*. *robur*

Response	Spring warming (W)			Genotype (G)			W × G			*A. brongniardellus*			Tree damage status		
	df	χ^2^/LR/F	*p*	df	χ^2^/LR/F	*p*	df	χ^2^/LR/F	*p*	df	χ^2^/LR/F	*p*	df	χ^2^/LR/F	*p*
Leaf development 8th May	1	190.61	**<.01**	5	33.30	**<.01**	5	9.46	.09	–	–	–	1	5.39	.**02**
Leaf development 11th May	1	213.76	**<.01**	6	29.67	**<.01**	6	5.82	.44	–	–	–	1	21.23	**<.01**
Chlorophyll content 30th August	1, 131	10.00	**<.01**	6, 131	6.66	**<.01**	6, 131	5.74	**<.01**	1, 131	0.11	.74	1, 131	7.81	.**01**
Chlorophyll content 12th September	1, 129	6.99	**<.01**	6, 129	4.83	**<.01**	6, 129	4.08	**<.01**	1, 129	1.70	.19	1, 129	3.96	.**05**
Chlorophyll content 19th September	1, 132	2.84	.09	6, 132	4.84	**<.01**	6, 132	3.16	**<.01**	1, 132	1.81	.18	1, 132	11.09	**<.01**
Chlorophyll content 1st October	1, 131	0.40	.53	6, 131	3.80	**<.01**	6, 131	2.03	.07	1, 131	6.98	.**01**	1, 131	8.91	**<.01**
Leaf coloration 1st October	1	0.50	.48	6	17.76	.**01**	C	C	C	1	2.01	.16	1	1.51	.22

Note

C – Not analysed due to convergence problems. For autumn phenology, we also estimate the effect of *A*. *brongniardellus* infestation. Spring phenology is based on a cumulative link mixed model, while chlorophyll content and leaf coloration are based on a linear and generalized linear model, respectively. Shown are likelihood ratio test for ordinal analyses and marginal or type 3 ANOVA of fixed effects. Significant *p*‐values (*p* < .05) are shown in bold.

### 3.2 Effect of spring warming on autumn phenology

**FIGURE 3:**
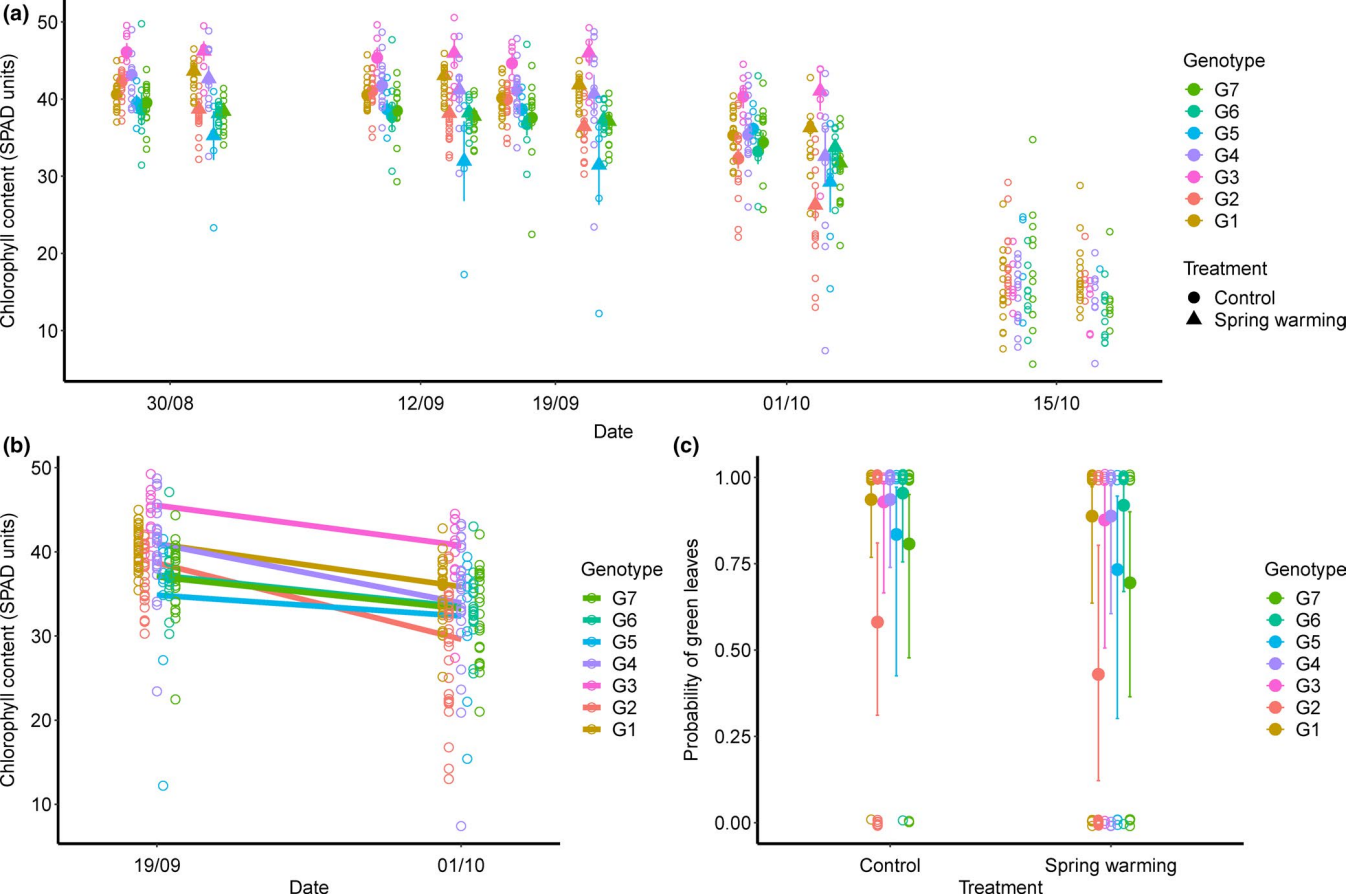
Autumn phenology of seven oak genotypes shown as (a) the chlorophyll content at five dates in autumn (*x*‐axis) on oaks subjected to different temperature treatments in spring. Shown are fitted values from four date‐specific models with standard errors, (b) the chlorophyll content at two dates in autumn (19th of September and 1st of October), and (c) the probability of oaks subjected to different temperature treatments in spring to have green leaves at October 1, modeled with 95% confidence intervals. Each color represents an oak genotype. Raw data is presented in the background as the average chlorophyll content on a leaf per oak (a, b) and the probability that leaves on an oak are green (c; jittered horizontally and vertically). Shown are fitted values from linear mixed models (a, b) and generalized linear mixed model (c)

On all four survey dates in autumn, chlorophyll content (signaling autumn coloration) differed among genotypes, whereas the size of this genotypic effect differed between temperature treatments (a significant Treatment x Genotype interaction; Table 1; Figure 3a). We found some evidence of an effect of spring warming on the autumn phenology: On October 1, when oaks leaves had started to loose chlorophyll, the Treatment x Genotype interaction suggests a more rapid loss of chlorophyll for genotype “G2” which also tended to develop buds early in the season (compare Figures 2 and 3a). Chlorophyll content in oak leaves declined between September 19 and October 1 (*F*_1, 144_ = 156.32, *p* < .01; Figure 3b) but the rate of chlorophyll loss differed among genotypes (interactions Genotype × Date; *F*_6, 144_ = 10.50, *p* < .01; with a main effect of genotype *F*_6, 139_ = 10.51, *p* < .01; Figure 3b). Oaks infested by high levels of *A*. *brongniardellus* earlier in the season were characterized by higher chlorophyll contents in the autumn (*F*_1, 139_ = 16.89, *p* < .01)—a pattern also brought out in the date‐specific models on October 1 (Table 1). Similarly, damaged trees were characterized by a higher chlorophyll content than were trees without damage (*F*_1, 139_ = 5.47, *p* = .02), which was also found in the date‐specific models (Table 1). For autumn leaf coloration on October 1, the probability of an oak having green leaves differed among genotypes, but not between temperature treatments (Table 1; Figure 3c).

### 3.3 Herbivore performance

**FIGURE 4:**
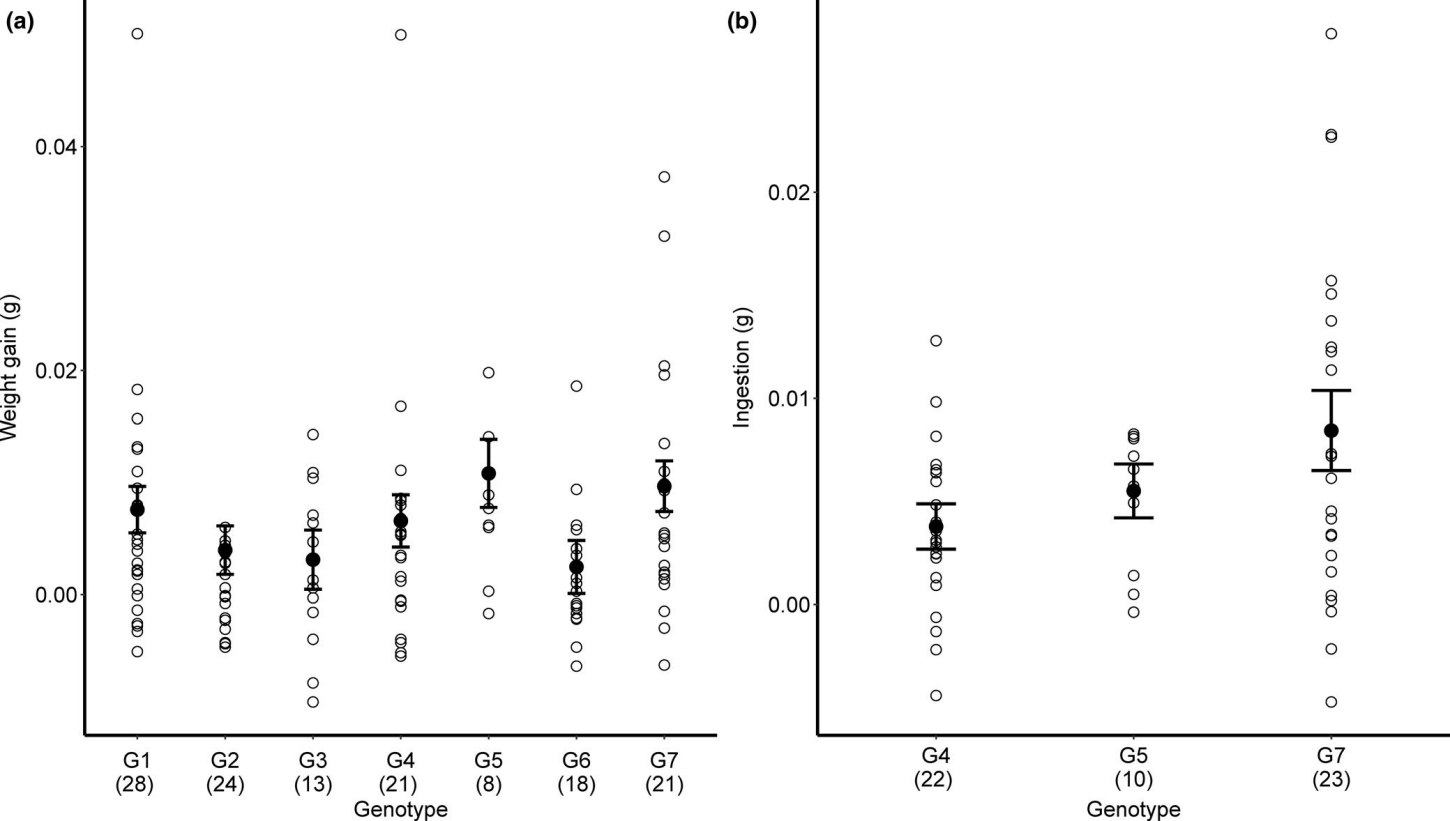
The effect of oak genotype on the (a) weight gain and (b) ingestion of two species of free feeding lepidopteran larvae. Shown are results from two feeding experiments that was conducted at June 6–8 with larvae of *Orthosia gothica* (a), and at August 21–23 with larvae of *Polia nebulosa* (b). Shown are fitted values from a linear mixed model with standard errors and raw data

In neither feeding experiments was there any detectable effect of plant phenology on herbivore performance (Table 2). In the first feeding experiment (June 6–8), the weight gain of *O*. *gothica* differed among broods and oak genotypes (Table 2; Figure 4), while ingestion was affected by the identity of the brood, and was higher on damaged trees (Table 2). In the second feeding experiment (August 21–23), we detected a near‐significant effect of traits associated with oak genotype on the ingestion of *P*. *nebulosa* (Table 2).

**TABLE 2:** The effect of spring warming (W), oak genotype (G), brood, tree damage status, and initial larvae weight on the weight gain and ingested leaf dry mass of *Orthosia gothica* and *Polia nebulosa*

Exp. Date/Response	Spring warming (W)			Genotype (G)			W × G			Tree damage status			Initial larval weight			Brood		
	df	χ^2^/F	*p*	df	χ^2^/F	*p*	dF	χ^2^/F	*p*	df	χ^2^/F	*p*	df	χ^2^/F	*p*	df	χ^2^/F	*p*
Exp I: June 6–8																		
Weight gain, *O*. *gothica*	1	0.72	.39	6	12.46	.05	6	3.44	.75	1	2.54	.11	1	2.04	.15	7	27.26	**<.01**
Ingestion, *O*. *gothica*	1, 112	0.49	.49	6, 112	1.11	.36	6, 106	0.73	.62	1, 112	4.05	.**05**	1, 112	10.55	**<.01**	7, 112	5.25	**<.01**
Exp II: August 21–23																		
Weight gain, *P*. *nebulosa*	1	0.20	.66	2	4.16	.12	2	1.98	.37	1	0.15	.69	1	0.19	.66	1	0.07	.79
Ingestion, *P*. *nebulosa*	1, 44	2.41	.13	2, 44	2.85	.07	2, 42	1.41	.25	1, 44	0.21	.65	1, 44	0.18	.67	1	0.15	.70

Note

The experiments have been conducted at two specific dates during a period of 48 h. Shown are results from a marginal or type 3 ANOVA of fixed effects of fixed effects in linear mixed models, with significant *p*‐values (*p* < .05) identified in bold.

### 3.4 Pathogen infection

Plant phenology and traits associated with oak genotype did not influence *E*. *alphitoides* incidence on any of the dates. Instead, oaks with high densities of *A*. *brongniardellus* had a lower chance of infection by *E*. *alphitoides* on June 30 (Table 3).

**TABLE 3:** The effect of spring warming (W), oak genotype (G), *A*. *brongniardellus* infestation, and tree damage status on the probability of *E*. *alphitoides* incidence at the tree level on five specific dates

*E*. *alphitoides* incidence at the tree level	Spring warming (W)			Genotype (G)			W × G			*A. brongniardellus*			Tree damage status		
	df	χ^2^	*p*	df	χ^2^	*p*	df	χ^2^	*p*	df	χ^2^	*p*	df	χ^2^	*p*
30th of June	1	0.17	.68	6	8.29	.22	C	C	C	1	6.37	.**01**	1	2.31	.13
16th of July	1	0.19	.66	6	5.76	.45	C	C	C	1	0.29	.59	1	0.59	.44
3rd of August	1	2.35	.13	6	9.84	.13	6	3.44	.75	1	0.44	.51	1	0.21	.65
27th of August	1	0	1	6	10.75	.10	6	3.34	.77	1	0.53	.46	1	2.13	.14
16th of September	1	1.17	.28	6	2.90	.82	6	2.69	.85	1	0.06	.80	1	0.27	.61

Note

C—Not analyzed due to convergence problems. Shown are results from a type 3 ANOVA of fixed effects in generalized linear mixed models, with significant *p*‐values (*p* < .05) identified in bold.

## 4 DISCUSSION

Leaf age and traits associated with plant genotype are regarded as important determinants for the performance of plant attackers (Barbehenn et al., 2017; Barker et al., 2019; Dodd et al., 2008; Edwards & Ayres, 1982; Ekholm et al., 2017; Falk et al., 2018; Tikkanen & Julkunen‐Tiitto, 2003). Therefore, predicting how plant phenology responds to elevated temperatures, and how this affects resource quality for higher trophic levels, requires an understanding of how climate warming influences plant phenology throughout the year. In this study, we found that warm spring temperatures advanced spring phenology. In addition, we found oak genotypes to differ in the timing of both spring and autumn phenology. Leaf age as such had no clear influence on the performance of plant attackers active in summer. Thus, our study shows that consumer species active in summer may be less affected by shifts in host plant phenology than what other studies have shown consumers active in spring and autumn to be (Ekholm et al., 2019; Falk et al., 2018; Tikkanen & Julkunen‐Tiitto, 2003). Below, we will discuss each finding in turn.

### 4.1 Temperature and plant phenology

We found that oaks exposed to elevated temperature in spring were more advanced in terms of bud development than were oaks exposed to ambient temperature. In addition, temperature treatment in spring influenced chlorophyll content in autumn, but not leaf coloration. On October 1, oaks had just initiated senescence and many of the leaves were still green and contained relatively high chlorophyll content. Our analysis of patterns recorded on this date showed that chlorophyll content differed between temperature treatments for at least one of the oak genotypes (“G2” in Figure 3a). Thus, we infer that only the oak genotypes of the earliest autumn phenology, and as subjected to the phenology‐advancing spring warming treatment, had started to senescence at this date. In consequence, we suggest that a survey slightly later in the season, when all genotypes had initiated senescence, might have resolved into a clearer pattern. However, the current indication of an advance in autumn phenology with a warming in spring is supported by other studies (Fu et al., 2014; Zohner & Renner, 2019), and thereby adds to extant evidence.

Phenology differed among genotypes in both spring and autumn phenology, where one genotype (“G2”, Figures 2 and 3) tended to be early in both spring and autumn. This suggests that a consumer species advancing its phenology at a rate similar to that of its host plant will maintain its interaction with specific genotypes, whereas shifts in phenological synchrony will shift the interaction strength. However, in this study, performance of plant attackers was insensitive to plant phenology.

### 4.2 Plant phenology and the performance of plant attackers

Several studies to date have shown that plant phenology can influence the performance of plant attackers (Barbehenn et al., 2017; Dodd et al., 2008; Edwards & Ayres, 1982; Falk et al., 2018; Tikkanen & Julkunen‐Tiitto, 2003). In our study, we explicitly focused on herbivore performance rather than chemical metrics, testing whether effects of plant spring phenology extend to consumers active slightly later in the season—on which we detected no effects of spring phenology. By the time of our feeding experiments, we find it likely that the chemical composition of the leaves had reached a phase of relatively stable chemical contents (Figure 1; see Salminen et al. (2004)). To this, we should add the fact that individual leaves within trees vary substantially in quality at any stage of the season (Gripenberg et al., 2007; Roslin et al., 2006), as also reflected by wide variation in any metric of performance scored here (see Figure 4). Hence, differences in leaf quality within specific time periods is likely to be larger than differences between periods.

As with the herbivores, we found no impact of plant phenology on incidence of *E*. *alphitoides* infection. Nonetheless, there is previous evidence to suggest such an effect: *E*. *alphitoides* colonizes oak leaves through ascospores, and the release of ascospores is related to temperature (Marcais et al., 2009). Thus, oaks, which spread their leaves before ascospores are released, have been shown to escape infection (Dantec et al., 2015). In our study system, the release of ascospores may have occurred already by the time the early phenology trees developed their leaves, thereby precluding any difference in exposure between oaks in the two temperature treatments.

Overall, the lack of effect on summer‐time plant consumers may reflect stability in leaf attributes during this period. Once again, we emphasize that with leaf attributes, we explicitly refer overall “leaf quality” integrated as herbivore performance, rather than variation in a myriad of physicochemical attributes of the leaf (for analyses at the latter level, see e.g., Salminen et al. (2004)). In the autumn, differences in such quality may again be accentuated, when leaves starts to senesce and nutrients are withdrawn (Maillard et al., 2015). Our study thus explicitly suggests that in between spring and autumn, variation in leaf age will have little effect on the performance of plant consumers (Figure 1).

### 4.3 Plant genotype and the performance of plant attackers

Traits associated with a specific genotype had an effect on insect herbivores but not on *E*. *alphitoides*. The findings from *E*. *alphitoides* differs from those reported by Roslin et al. (2007), who detected pronounced differences in infection levels between replicate samples of foliage from the same tree, and differences in performance between *E*. *alphitoides* strains from the original host oak and other trees. Also Ekholm et al. (2017) found substantial differences in infection between oak genotypes. In this experiment, we detected no effect of traits associated with genotype on *E*. *alphitoides* performance—potentially because there are several strains of *E*. *alphitoides* present in the region, in which case each oak genotype can be attacked by at least some strains of *E*. *alphitoides*. Such a pattern is consistent with the high overall infection rates detected by Roslin et al. (2007), despite pronounced differences in performance among strains on individual oak genotypes.

In terms of insect performance, we found that traits associated with plant genotype influenced performance: in the first feeding experiment (June 6–8), we found a difference in the weight gain of *O*. *gothica* among oaks of different genotype and in the second feeding experiment (August 21–23), we detected a near‐significant effect of oak genotype on the ingestion of *P*. *nebulosa*. In the former case, we found mean performance to differ by a factor of 4.38 between the best and worst genotype, whereas in the latter case the factor was 2.23. Generalist species like *O*. *gothica* and *P*. *nebulosa* can be expected to be sensitive to traits associated with plant genotype since the performance of generalists varies more than for specialists (Barker et al., 2019). In addition, specialist herbivores sometimes deal better with plant defenses than generalists (Roslin & Salminen, 2008). Importantly, we observed large variation within each genotype in each metric of herbivore performance (Figure 4). This is likely to reflect large inherent variation among leaves and branches within genotypes (Gripenberg et al., 2007; Roslin et al., 2006). Thus, the lack of differences in larval performance among trees subjected to different treatments, and among traits associated with genotype seem more indicative of the real lack of biologically relevant effects than the lack of statistical power. Compared to the large variation inherent in larval performance, and large variation in foliage between different parts of a tree, the imprint of spring‐time temperature is biologically secondary.

## 5 CONCLUSION

As our most important finding, our study fills in the previous knowledge gap of phenological impacts on plant attackers feeding in early spring and late autumn, suggesting that consumer species of intermediate phenological occurrence may be less affected by shifts in host plant phenology. We also found that warmer springs caused an advance in phenology, and that this effect carried over to the autumn, when early bud break in the spring was associated with early leaf senescence in the autumn. Yet, these shifts in the start and the end of leaf life span had little effect on herbivores feeding on leaves in summer.

To conclude, plant attackers who are active in summer might be more tolerant to temperature‐induced shifts in host plant phenology than to what has been previously documented for plant attackers that are active in spring and autumn. Clearly, more research will be needed on more species that are finely spaced along the spring‐autumn continuum, but this study provides a first glimpse of the patterns in mid‐season.

## CONFLICT OF INTEREST

They have no conflict of interest to declare.

## AUTHOR CONTRIBUTIONS

**Adam Ekholm:** Conceptualization (equal); Formal analysis (equal); Investigation (lead); Methodology (equal); Visualization (equal); Writing – original draft (lead); Writing – review & editing (equal). **Maria Faticov:** Conceptualization (equal); Investigation (equal); Methodology (equal); Visualization (supporting); Writing – review & editing (equal). **Ayco J. M. Tack:** Conceptualization (equal); Methodology (equal); Supervision (supporting); Writing – review & editing (equal). **Tomas Roslin:** Conceptualization (equal); Formal analysis (supporting); Funding acquisition (lead); Methodology (equal); Resources (equal); Supervision (lead); Writing – review & editing (equal).

## Supporting information

Appendix S1

## Data Availability

Data available from the Dryad Digital Repository: https://doi.org/10.5061/dryad.9ghx3ffjs.
