# Supplementary material for: Herbivory in a changing climate—Effects of plant genotype and experimentally induced variation in plant phenology on two summer‐active lepidopteran herbivores and one fungal pathogen
Source: Ecol Evol. 2022 Jan 25;12(1):e8495. doi: 10.1002/ece3.8495 (PMC8796927; doi:10.1002/ece3.8495)
Supplement: Supplementary file 1 — Appendix S1 [file ECE3-12-e8495-s001.docx]

**Supporting information**

**Appendix A.** Supplementary tables

Table S1. The number of oaks used to analyse spring and autumn phenology.

Table S2. Scale used for scoring phenology at the shoot level.

Table S3. A summary of the statistical models fitted in this study

Table S4. Scale used for scoring phenology at the tree-level.

**Appendix B.** Controlling for effects of temperature treatments applied in the previous year.

**Table S1.** The number of oak genotypes that was used to analyse spring and autumn phenology and their representation in each block. Each column represents a genotype, while each row represents a block from one of the two analyses.

| Analysis/Block | G1 | G2 | G3 | G4 | G5 | G6 | G7 |
| --- | --- | --- | --- | --- | --- | --- | --- |
| Spring phenology |  |  |  |  |  |  |  |
| 1 | 6 | 6 | 2 | 5 | 2 | 4 | 4 |
| 2 | 7 | 6 | 2 | 5 | 2 | 4 | 6 |
| 3 | 7 | 6 | 4 | 5 | 2 | 5 | 5 |
| 4 | 6 | 7 | 4 | 7 | 2 | 5 | 4 |
| 5 | 6 | 5 | 3 | 4 | 3 | 4 | 5 |
| Autumn phenology |  |  |  |  |  |  |  |
| 1 | 6 | 6 | 2 | 5 | 2 | 4 | 4 |
| 2 | 7 | 6 | 1 | 5 | 1 | 3 | 6 |
| 3 | 7 | 6 | 3 | 4 | 2 | 4 | 5 |
| 4 | 6 | 7 | 4 | 7 | 1 | 5 | 4 |
| 5 | 6 | 5 | 3 | 4 | 3 | 4 | 5 |

**Table S2.** The phenological scale used to score spring phenology on the shoot-level, following Hinks et al. (2015).

| Stage | Description |
| --- | --- |
| 1 | Small dormant buds |
| 2 | Large, slightly elongated buds |
| 3 | Larger, loosened greenish brown buds |
| 4 | Elongated buds with leaves starting to erupt (i.e., bud burst) |
| 5 | The first leaves leave the bud casings, separate and begin to spread out |
| 6 | Leaves emerging but still tight |
| 7 | Leaves are fully expanded and adopt their mature, dark green coloration |

**Table S3.** A summary of the models fitted in this study. The first column refers to the specific model that is specified in the statistical methods section and the second column to the modelling framework (lm = linear model, lmm = linear mixed model, glmer = generalized linear mixed model) and the data distribution. The columns that follow show the response variable, explanatory variables, random effects and explanatory variables with separate variance structures in each model.

| **Model** | **Framework / Distribution** | **Response variable** | **Explanatory variables** | | | | | | **Random effect** | | **Variance structure** |
| --- | --- | --- | --- | --- | --- | --- | --- | --- | --- | --- | --- |
| 1 | clmm / Multinomial | Shoot level phenology | Genotype | Treatment | Genotype x Treatment | Tree damage status | - | - | | Block/Oak | - |
| 2 | lmm / Normal | Chlorophyll content* | Genotype | Treatment | Genotype x Treatment | Tree damage status | *A. brongniardellus* | - | | Block | Genotype x Treatment |
| 3 | lmm / Normal | Chlorophyll content | Genotype | Date | Genotype x Date | Tree damage status | *A. brongniardellus* | - | | Block/Oak | Genotype, Tree damage status |
| 4 | glmer / Binomial | Autumn leaf coloration | Genotype | Treatment | Tree damage status | *A. brongniardellus* | - | - | | Block | - |
| 5 | lmm / Normal | Weight gain, *O. gothica* | Genotype | Treatment | Genotype x Treatment | Brood | Initial larval weight | Tree damage status | | Block | - |
| 6 | lmm / Normal | Ingestion, *O. gothica* | Genotype | Treatment | Genotype x Treatment | Brood | Initial larval weight | Tree damage status | | Block | Tree damage status |
| 7 | lmm / Normal | Weight gain, *P. nebulosa* | Genotype | Treatment | Genotype x Treatment | Brood | Initial larval weight | Tree damage status | | Block | - |
| 8 | lmm / Normal | Ingestion, *P. nebulosa* | Genotype | Treatment | Genotype x Treatment | Brood | Initial larval weight | Tree damage status | | Block | Genotype |
| 9 | glmer / Binomial | Mildew Incidence | Genotype | Treatment | Genotype x Treatment | Tree damage status | *A. brongniardellus* | - | | Block | - |
| 10 | lm / Normal | Spring phenology 2018 | Genotype | Treatment | Autumn phenology 2017 | Spring phenology 2017 | Temperature treatment 2017 | Tree damage status | | - | Tree damage status |
| * Modelled separately at four dates (August 30, September 12, September 19 and October 1) | | | | | | | | | | | |

**Appendix B.** Controlling for effects of temperature treatments applied in the previous year.

One year prior to the current experiment (year 2017), we studied oak phenology on 117 of the 160 grafted oaks (5 genotypes). This was done in a heating experiment, where 59 oaks were subjected to an experimental warming of ca 2 °C with ceramic infrared heaters for a full growing season *in situ*, while 58 oaks were exposed to ambient temperature. All trees were divided into six mesh net cages (5 x 5 x 2.2 m), out of which three were experimentally warmed with infrared heaters. In the spring and autumn of 2017, we scored leaf development (median leaf development stage at five shots) and leaf senescence. Leaf senescence was defined as the day when 50% of the tree leaves had turned brown, interpolated from the measures of leaf senescence between two successive observations (Faticov et al. 2020) (see fig. S1 for temperature treatments in 2017 and 2018).

To assess whether spring phenology was influenced by autumn phenology in the previous year and whether the rank among oaks in spring phenology differed between years, we modelled spring phenology at the tree level (the Julian day a tree had reached stage 19 on a modified BBCH-scale (Meier 2001), table S4) as a function of temperature treatment in 2017 and spring and autumn phenology in 2017. To control for the effect of genotype, tree damage status and spring warming in 2018, we also included these as fixed effects in the model (these variables are also analysed at the level of shoots in the main manuscript). To account for variation among blocks, we included block as a random effect. As the fixed effect Tree damage status differed in variance among the two groups (i.e violated the assumption of homogeneity of variance), we fitted a separate variance to each level of tree damage status (model 10 in table S4).

Our analyses revealed consistency in tree-level spring phenology between years: Spring phenology in year 2017 was related to spring phenology in year 2018 (F_1, 103_ = 3.64, p = 0.06). However, autumn phenology in year 2017 did not predict spring phenology in year 2018 (F_1, 103_ = 0.44, p = 0.50), neither did we detect any effect of the temperature treatment applied in year 2017 on spring phenology in 2018 (F_1, 103_ = 1.31, p = 0.26). Trees with no damage (F_1, 103_ = 10.53, p > 0.01) and oaks subjected to spring warming (F_1, 103_ = 247.18, p > 0.01) tended to be earlier in their spring phenology in year 2018. In addition, spring phenology differed among oak genotypes (F_4, 103_ = 5.38, p > 0.01). Overall, we were thus able to reject the notion that temperature treatments applied during previous years might have affected the tree-level responses observed in the current experiment.

**Figure S1.** The temperature treatments that the oaks have been subjected to during two years. In 2017, 59 oaks were exposed to *in situ* experimental warming by ceramic infrared heaters from May until October, while 58 oaks were kept as controls and exposed to ambient temperature. In 2018, 77 oaks were exposed to spring warming in a greenhouse from late March to early May, while 83 oaks were exposed to ambient temperature.

**
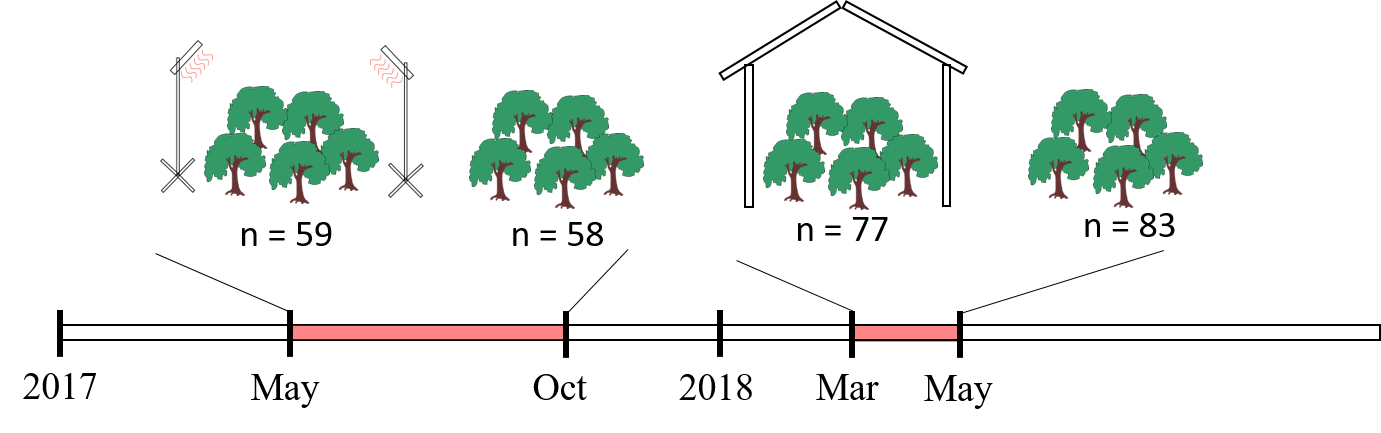
**

**Table S4.** The phenological scale used to score bud development on the tree-level in spring following a modified version of the international phenological scale (Meier 2001).

| Stage | Description |
| --- | --- |
| 0 | Period of winter dormancy – buds are closed and covered in brown scales |
| 1 | The majority of leaf buds have started to expand – their scales lengthen and buds become slightly paler at the tips |
| 3 | The majority of leaf buds have finished expanding and are ready to open – they become more rounded at the tips |
| 9 | The tips of the first leaves are clearly visible and extend past the end of the bud scales |
| 10 | The first leaves leave the bud casings, separate and begin to spread out |
| 12 | The first leaves are spread out over 50% of the crown |
| 19 | The first leaves are spread out over 90% of the crown |

**References**

Faticov, M., et al. 2020. Climate and host genotype jointly shape tree phenology, disease levels and insect attacks. - Oikos 129: 391-401.

Hinks, A. E., et al. 2015. Scale-Dependent Phenological Synchrony between Songbirds and Their Caterpillar Food Source. - American Naturalist 186: 84-97.

Meier, U. (ed.). 2001. Growth stages of mono-and dicotyledonous plants, BBCH Monograph. - Federal Biological Research Centre for Agriculture and Forestry.
